# Editorial: Hazardous pollutants in agricultural soil and environment

**DOI:** 10.3389/fmicb.2024.1411735

**Published:** 2024-04-24

**Authors:** Reeta Goel, Pankaj Chaturvedi, Saurabh Kumar, Ravindra Soni, Deep Chandra Suyal

**Affiliations:** ^1^Institute of Applied Sciences and Humanities, GLA University, Mathura, India; ^2^Department of Cell and Developmental Biology, University of Illinois at Urbana-Champaign, Urbana, IL, United States; ^3^Indian Council of Agricultural Research (ICAR)-Research Complex for Eastern Region, Patna, India; ^4^Department of Agricultural Microbiology, College of Agriculture, Indira Gandhi Krishi Vishwa Vidyalaya, Raipur, India; ^5^Vidyadayini Institute of Science, Management, and Technology, Bhopal, India

**Keywords:** heavy metal, sustainable agriculture, pollution, biohazard, microorganisms

Food security is a cornerstone of global health, human wellbeing, and economic stability. Ensuring the availability, accessibility, and quality of food is fundamental for sustaining populations worldwide, but this critical need is threatened by contaminants that diminish agricultural productivity and compromise food security. These pollutants, including heavy metals and metalloids, persistent organic pollutants, pesticides, and emerging threats like microplastics and nanoplastics, not only disrupt agricultural productivity but also pose significant risks to environmental sustainability, and human health.

The detrimental effects of soil contamination on agricultural outputs are multifaceted. Heavy metals such as lead and cadmium can severely hinder plant growth, reduce crop yields, and compromise the nutritional quality of the food produced (Kumar et al., 2021; Madhav et al., 2024). Arsenic on the other hand is a toxic metalloid that has been reported as the major determinant for decreasing grain yield besides causing straighthead disease in rice (Kumar et al., 2022). Similarly, the widespread use of chemical pesticides and herbicides, while aiming to enhance crop protection, often leads to long-term soil degradation and loss of biodiversity, further diminishing the soil's natural capacity to support agriculture. The infiltration of these toxic substances into soils, a direct consequence of industrial activities, agricultural practices, and inadequate waste management, leads to a cascade that disrupts the delicate balance of nutrients and microbial life critical for plant growth and soil health (Swain, 2024). This xenobiotic contamination undermines the nutritional value of crops and raises the chances of bioaccumulation of harmful chemicals in the human body. This can potentially lead to chronic health conditions in humans, including cancers, neurological disorders, and developmental issues in children. The challenge is further compounded by the emerging threat of microplastics, whose long-term ecological and health impacts are only beginning to be understood. Therefore, both, food safety and security are compromised due to the presence of toxic contaminates in agricultural soil.

Addressing the challenges posed by soil pollution to food security requires a concerted effort to implement sustainable agricultural practices, innovative remediation technologies, and robust policies aimed at protecting soil health, thereby ensuring the production of safe, nutritious food and safeguarding public health for future generations (Goel et al., 2021). In this direction, the current Research Topic highlights the problems associated with hazardous pollutants in agricultural soil and recent advancements to tackle their remediation. It further aims to spotlight the critical issues surrounding soil pollution and showcase recent breakthroughs in pollution remediation for agricultural soil. This Research Topic includes 13 original research articles and one review article.

Chang et al. demonstrate the hazards posed by the commonly used fungicide (dimethomorph) and pesticide (imidacloprid) in Taiwanese vineyards. Through orthogonal approaches of microbial population analysis, the authors reveal alteration in soil bacterial composition leading to an inhibitory effect on soil metabolism. Notably, imidacloprid is banned by the European Union due to its toxicity for bees and wild pollinators (https://www.science.org/content/article/european-union-expands-ban-three-neonicotinoid-pesticides). Reiß et al. observed the similar results by studying the microbial composition of honeybee (*Apis mellifera*) cuticles. Both studies conclude that the use of fungicides alone or in combination with other pesticides can alter microbiome composition. Similar impacts of xenobiotics on microbial diversity have been reported in this Research Topic by Gangola et al. through 16S rDNA-based metagenomic analysis of pesticide-contaminated soil.

An appropriate combination of xenobiotics and organic farming approaches can mitigate this loss of microbial diversity and maintain the bioremediation potential of the soil microbiota. This idea is supported by the observations reported by Colautti et al., where the authors compared the organically vs. conventionally managed vineyards. Emerging solutions, such as the use of bio-inoculants and leguminous plants for the remediation of heavy metal-contaminated soils, offer promise for restoring soil health and reducing pollutant bioavailability. The work of Zheng et al., for example, provides evidence supporting the efficacy of such bioremediation strategies in dealing with cadmium and lead contamination. Additionally, research into soil amendment techniques, including composting and the use of chelators like EDTA, as reported by Xu et al. and Kamal et al., respectively, suggests practical methods for improving soil conditions and mitigating pollutant impacts. Kumar et al., further emphasized the role of exogenously applied brassinosteroids in phytoremediation projects by ameliorating heavy metal stress. Avatsingh et al., highlighted the concerns on the presence of antibiotic-resistant bacteria in polluted irrigation-purpose wastewaters. Debbarma et al., explored the potential of microbial bioformulation (monoculture *Pseudomonas aeruginosa* strain PE10) for e-waste bio-recycling in agricultural soil ecosystems, thus managing the e-waste crisis in agricultural soil through sustainable ways.

Conclusively, this Research Topic confers useful updates and advancements about the existence of hazardous pollutants in the agricultural fields. The collaborative efforts of researchers, farmers, policymakers, and the broader public are imperative in this endeavor, highlighting the collective responsibility to preserve the foundation of food security for generations to come. We have a firm believe that these studies, along with sustainable agricultural practices and policy reforms, will be useful in combating soil pollution, ensuring the sustainable future of agriculture, and protecting public health.

## Author contributions

RG: Project administration, Supervision, Writing – review & editing. PC: Writing – original draft, Writing – review & editing. SK: Conceptualization, Writing – original draft, Writing – review & editing. RS: Writing – review & editing. DS: Writing – review & editing.
